# TMAO: a potential mediator of clopidogrel resistance

**DOI:** 10.1038/s41598-021-85950-8

**Published:** 2021-03-22

**Authors:** Ruisong Ma, Wenwen Fu, Jing Zhang, Xiaorong Hu, Jun Yang, Hong Jiang

**Affiliations:** 1 Department of Cardiology, Hainan General Hospital, Haikou, People’s Republic of China; 2 Department of Cardiology, Renmin Hospital of Wuhan University, Wuhan University, Wuhan, People’s Republic of China; 3 Department of Cardiology, The First College of Clinical Medical Sciences, China Three Gorges University, Yichang, People’s Republic of China; 4 Department of Cardiology, Zhongnan Hospital of Wuhan University, Wuhan University, Wuhan, 430071 People’s Republic of China

**Keywords:** Platelets, Acute coronary syndromes

## Abstract

Trimethylamine-N-oxide (TMAO) can activate platelets and increase thrombosis risk in clinical and experimental models. Meanwhile, the patients with coronary artery disease have higher serum TMAO level. However, it remains unknown whether Clopidogrel Resistance (CR) could be attributed to TMAO. The present study aimed investigate the effects of TMAO on clopidogrel in ischemia and reperfusion (IR) model in rats. Clopidogrel could (1) promote the production of platelets, induce an increase in the platelet-larger cell ratio; (2) prolong the tail bleeding time; (3) reduce platelet aggregation function, induced by ADP, and alleviate myocardial thrombus burden. TMAO could partially offset the effects of clopidogrel and induce CR. Thus, the present study demonstrated that circulating TMAO could reduce the inhibitory effects of clopidogrel on platelet aggregation. TMAO may be a potential mediator of clopidogrel resistance.

## Introduction

Dual antiplatelet therapy (DAPT) with aspirin and clopidogrel has been the cornerstone of the treatment of acute coronary artery syndrome (ACS) and consequent percutaneous coronary intervention for decades. However, the high rate of clopidogrel resistance (CR), which can be as high as 40%^1^, restricts its clinical curative effects, and the latest ESC guidelines recommend the limited use of clopidogrel^2^. However, clopidogrel is still the most commonly prescribed antiplatelet drug^3^. This might be attributable to its wide acceptance, low cost, good safety and low bleeding risk. An overwhelming majority of investigations have focused on the pharmacokinetics of clopidogrel by investigating the CYP2C19 gene polymorphism and treatment with calcium channel blockers^4–7^. Previous studies demonstrated that genetic polymorphisms in CYP2C19 contributed to CR (approximately 5% to 12%) but did not fully explain the occurrence of CR in most patients or in patients with wild-type genes^8–10^. In addition, although the metabolism of clopidogrel and prasugrel depends on CYP2C19, CYP2C19 loss-of-function alleles had no significant effect on the antiplatelet effects of prasugrel^11^. There were few reports of prasugrel resistance. Thus, pharmacodynamic factors should be given more attention.

TMAO, a waste product of gut microbes that is oxidized by flavin monooxygenases in the liver^12^, has been proven to accelerate the development of coronary artery disease and enhance platelet responsiveness and thrombosis risk^12,13^. Patients with coronary artery disease have higher circulating TMAO levels^14^. Elevated serum TMAO levels are independently associated with coronary artery disease development and have predicted an increased thrombotic event risk in large-scale clinical studies^13,15,16^. Every 10 µmol/L increase in the TMAO level led to a 7.6% increase in all-cause mortality^17^. However, whether the hyperresponsiveness of platelets induced by TMAO could affect the antiplatelet activity of clopidogrel and whether the elevated TMAO level in CAD patients is a potential contributor to clopidogrel resistance remain unclear. In the present study, we will explore the above questions in an ischemia and reperfusion (IR) model in rats.

## Materials and methods

### ARRIVE guidelines and ethical approval of the study protocol

All experimental protocols in this study conformed to the Guidelines for the Care and Use of Laboratory Animals published by the US National Institutes of Health (NIH Publication, revised 1996) and were approved by the Animal Experimental Center of Renmin Hospital of Wuhan University. The study is also in compliance with ARRIVE guidelines.

### Animal preparation and experimental design

Nineteen male Sprague–Dawley rats (180–230 g, 6–8 weeks old) were randomly divided into three groups.

Group 1. IR group (n = 6): intragastric administration of normal saline (1 ml) for four days; rats were subjected to left descending coronary artery (LAD) occlusion for 30 min followed by reperfusion for 4 h. After being anesthetized, the rats were treated with normal saline (0.5 ml per rat, i.v. via the tail vein) 30 min before LAD occlusion.

Group 2. Clopidogrel + IR group (n = 7): Clopidogrel (1 mg/kg/day, Sanofi) powder suspended in normal saline (1 ml) was intragastrically administered for four days, and the rats were subjected to left descending coronary artery (LAD) occlusion for 30 min followed by reperfusion for 4 h. After being anesthetized, the rats were treated with normal saline (0.5 ml per rat, i.v. via the tail vein) 30 min before LAD occlusion.

Group 3. TMAO + clopidogrel + IR group (n = 6): Clopidogrel (1 mg/kg/day, Sanofi) powder suspended in normal saline (1 ml) was intragastrically administered for four days, and the rats were subjected to left descending coronary artery (LAD) occlusion for 30 min followed by reperfusion for 4 h. After being anesthetized, the rats were treated with TMAO (500 mg/kg rat weight^13^ dissolved in 0.5 ml normal saline, i.v. via the tail vein) 30 min before LAD occlusion.

The rats were anesthetized with 2.5% sodium pentobarbital (45 mg/kg, i.p.).

The IR model in rats was performed as previously described^18^.

After 4 h of reperfusion, rats were anesthetized with a half dose (22.5 mg/kg, i.p.) of 2.5% sodium pentobarbital. Two milliliters of blood (0.5 ml for routine blood tests, 1 ml for platelet aggregation tests, and 1 ml for platelet concentration) was collected from the jugular vein. Then, the heart was excised quickly. The infarct area (white) and risk area (5 mm around the infarct area) were retained and cut into two equal parts. After washing, one part was immediately frozen at -80 °C, and the other part was fixed with 4% paraformaldehyde for subsequent assays. The personnel who performed these assays were blinded to the treatment allocation.

### Routine blood testing

Blood (0.5 ml) was collected into a BD Vacutainer tube containing ethylenediaminetetraacetic acid disodium salt (EDTA). The platelet count, mean platelet volume (MPV) and platelet-larger cell ratio (P-LCR) were assessed (XN-9000, Sysmex Corporation, Japan).

### Platelet function

One milliliter of blood was collected into a BD Vacutainer tube containing acid citrate dextrose (ACD), and the tube was inverted 10 times to mix the contents, which were then filtered with a 300 mesh nylon screen (Beijing Solarbio Science & Technology Co., Ltd.). Adenosine diphosphate (ADP)-mediated platelet aggregation, including the maximum agglutination ratio (MaxAR-ADP), maximum agglutination time (MaxAT-ADP) and average agglutination ratio (AveAR-ADP), was detected (PL-12, SINNOWA, China). CR was defined as a decrease in the platelet aggregation rate induced by clopidogrel under ADP stimulation of at least 30% compared with the mean value in the IR group.

Tail bleeding time: After 4 h of reperfusion, the tail was cut off at a site 0.5 cm from the end. The tail stump was immediately placed in 0.9% NaCl solution at 36 °C, and the bleeding time starting from when the tail was placed in the salt solution until bleeding stopped was recorded. Bleeding times longer than 15 min were recorded as 15 min.

### Immunofluorescence staining

Briefly, after the sections were mounted and dewaxed, antigen retrieval was performed by a high-temperature, high-pressure method. After blocking in donkey serum (AntGene, ANT051), the sections were probed with primary antibodies, including anti-CD 41 antibody (diluted 1:500, Abcam, ab181582) and anti-CD 31 antibody (diluted 1:1000, Abcam, ab24590), overnight at 4 ℃. The sections were incubated with Alexa Fluor 488 donkey anti-rabbit lgG (diluted 1:400, Life Technologies, A21206) for 30 min at 37 ℃, and DAPI (1:500, Roche, 216276) was used to stain the nuclei. The platelets and thrombi were stained green and scanned by a fluorescence scanner (pannoramic Viewer MIDI II). Green foci with diameters less than 8 µm dismissed at suitable brightness and contrast in Pannoramic Viewer software. The maximum density (MD), defined as the maximum area of green foci in a fixed area of 750 µm × 750 µm at 100 × , and the thrombosis index (TI), defined as the ratio of the total green-stained area to the total stained area, were used to estimate the myocardial thrombus burden.

### Statistical analysis

SPSS 20.0 was used for all statistical analyses. Quantitative data are expressed as the mean ± SD or median ± quartiles. The Kolmogorov–Smirnov test was used to verify the normal distribution of data. Student’s t-test was used for between-group comparisons. One-way ANOVA was used to analyze comparisons among groups, and the LSD post hoc test or Tamhane’s T2 test was used for multiple comparisons. The Kolmogorov–Smirnov test was used for comparisons between groups with non-normally distributed data. A *P* value < 0.05 was considered statistically significant.

## Results

### Routine blood testing

**Figure 1:**
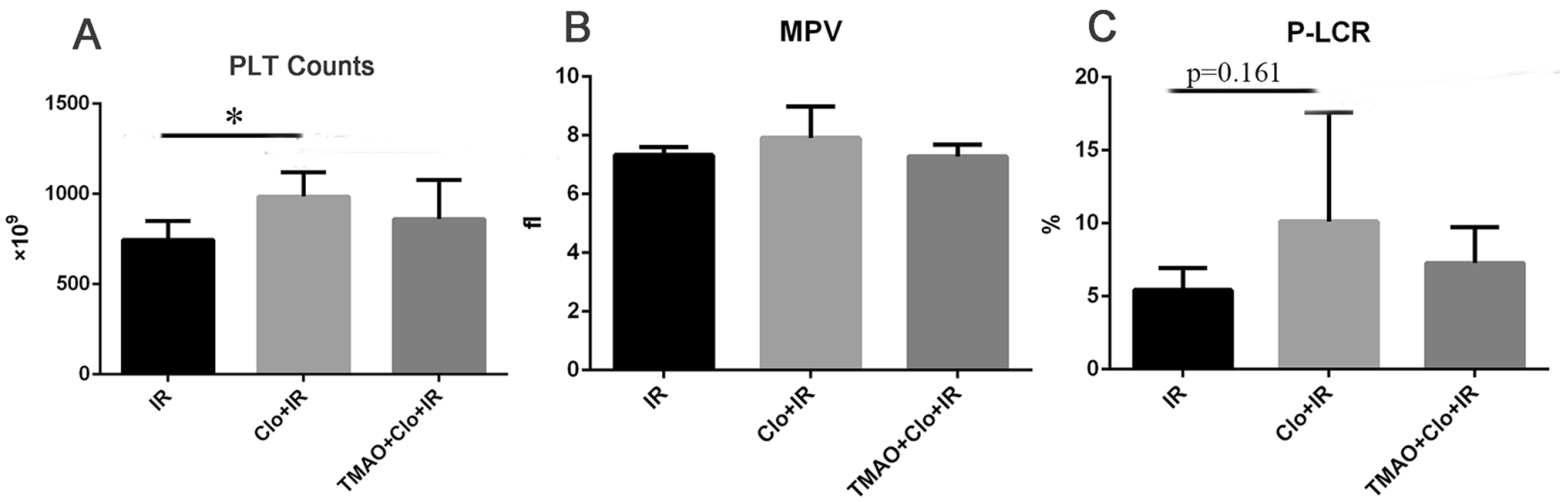
Clopidogrel induced the production of PLT and an increase in the P-LCR ratio. PLT: platelet; MPV: mean platelet volume; P-LCR: platelet larger cell ratio. *P < 0.05.

Compared with the IR group, the clopidogrel + IR group had a significantly larger PLT count (985.33 ± 133.51 × 10^9 pcs/L vs 745.50 ± 105.71 × 10^9 pcs/L, *P* < 0.05) and an increase in the P-LCR (10.13 ± 7.48% vs 5.42 ± 1.51%, *P* = 0.161), but there was no significant difference in the MPV (7.92 ± 1.06 fL vs 7.33 ± 0.27 fL, *P* > 0.05). Compared with the values for the clopidogrel + IR group, the addition of TMAO had no significant effect on the PLT count (861.17 ± 216.12 × 10^9 pcs/L, *P* > 0.05), P-LCR (7.28 ± 2.45%, *P* > 0.05) or MPV (7.28 ± 0.39 fL, *P* > 0.05) (Fig. 1).

### Tail bleeding time

**Figure 2:**
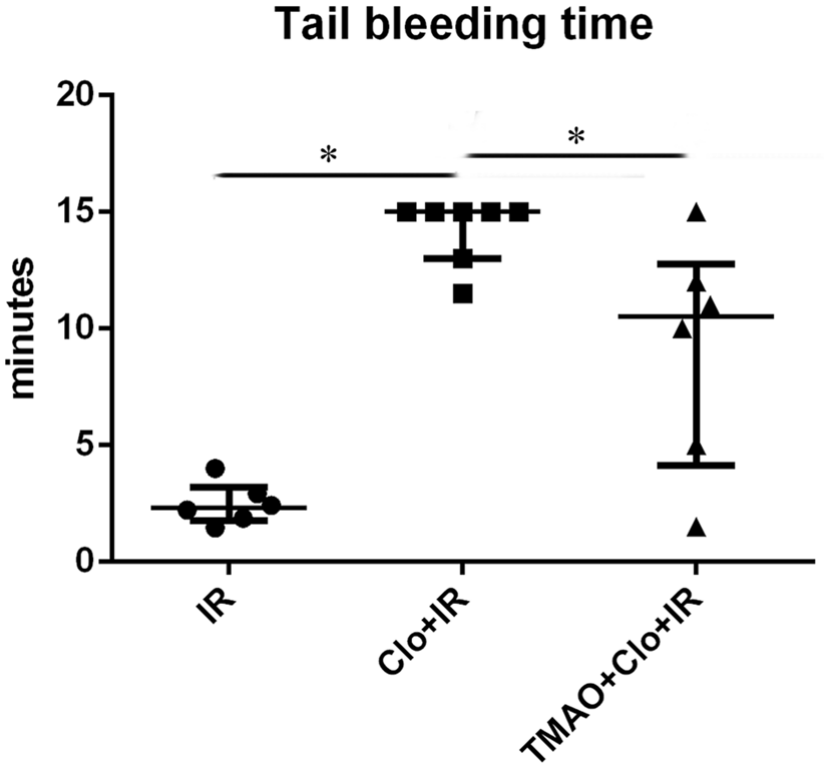
Clopidogrel prolonged the tail bleeding time, and this effect could be partially suppressed by TMAO. *P < 0.05.

Compared with the IR group, the clopidogrel + IR group had a significantly longer tail bleeding time (15.0 ± 2.37 min vs 2.33 ± 1.43 min, *P* < 0.05). Compared with that in the clopidogrel + IR group, the tail bleeding time showed a decreasing trend in the TMAO + clopidogrel + IR group (10.5 min ± 8.63 min, *P* = 0.139) (Fig. 2).

### Platelet aggregation

**Figure 3:**
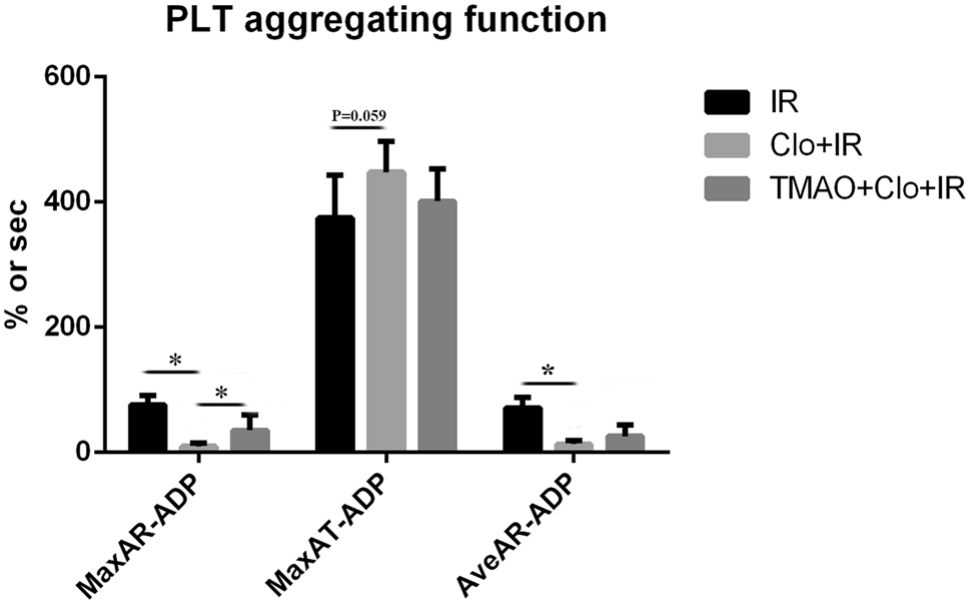
Clopidogrel inhibited platelet aggregation, and its effects could be partially suppressed by TMAO. *P < 0.05.

Compared with the IR group, the clopidogrel + IR group had lower MaxAR-ADP (9.65 ± 5.60% vs 75.52 ± 14.99%, *P* < 0.05) and AveAR-ADP values (13.22 ± 5.81% vs 71.32 ± 16.66%, *P* < 0.05) and showed an increasing trend for MaxAT-ADP (447.67 ± 49.45 s vs 374.83 ± 67.81 s, *P* = 0.059); the TMAO + clopidogrel + IR group had lower MaxAR-ADP (35.05 + 24.93%, *P* < 0.05) and AveAR-ADP values (25.50 + 18.60%, *P* < 0.05). Compared with the clopidogrel + IR group, the TMAO + clopidogrel + IR group had a significantly larger MaxAR-ADP value (35.05 + 24.93%, *P* < 0.05), an increasing trend for the AveAR-ADP value (25.50 + 18.60%, *P* = 0.171), and a similar MaxAT-ADP value (401.17 + 51.41 s, *P* > 0.05) (Fig. 3).

No CR was observed in the clopidogrel + IR group (0/7), and two cases with CR were observed in the TMAO + clopidogrel + IR group (2/6).

### Myocardial thrombosis staining

**Figure 4:**
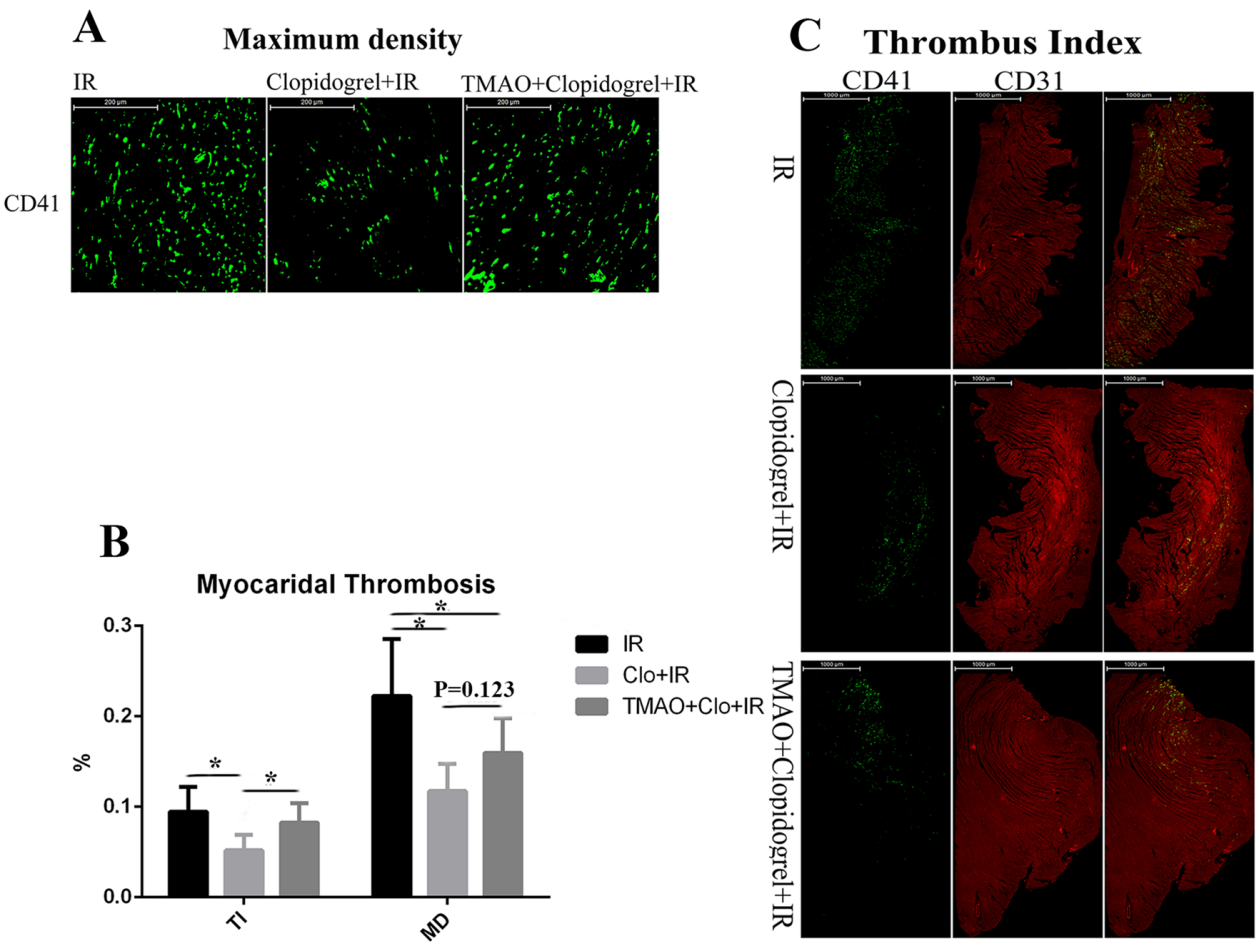
Clopidogrel reduced the myocardial thrombus burden and maximum density; TMAO partially increased the MD. *P < 0.05.

Compared with the IR group, the clopidogrel + IR group had smaller TI (0.052 ± 0.017% vs 0.096 ± 0.031%, *P* < 0.05) and MD values (0.12 ± 0.030% vs 0.22 ± 0.063%, *P* < 0.05); the TMAO + clopidogrel + IR group had smaller MD (0.16 ± 0.039%, *P* < 0.05) and TI values (0.083 ± 0.021%, *P* > 0.05). Compared with that in the clopidogrel + IR group, TMAO significantly increased the myocardial thrombus burden (TI: 0.083 ± 0.021%, *P* < 0.05; MD: 0.16 ± 0.039%, *P* = 0.123) (Fig. 4).

## Discussion

Clopidogrel, an antagonist of the platelet P2Y12 receptor, inhibits platelet aggregation by outcompeting ADP for binding of the P2Y12 receptor and subsequently activating glycoprotein IIb/IIIa. In the present study, we found that clopidogrel sufficiently inhibited platelet aggregation (Fig. 3), prolonged the tail bleeding time (Fig. 2) and reduced the myocardial thrombosis burden (Fig. 4). No CR was observed in the clopidogrel + IR group. Meanwhile, clopidogrel could induce the production of platelets and resulted in an increasing trend in the P-LCR ratio (Fig. 1). Normal circulating platelets have a size of 2–3 μm. Large platelets have a size over 6 μm and are commonly seen in reticulate platelets, also named newly-formed platelets. Therefore, the ratio of large platelets partly reflects the ability of platelet production. Compared to normal platelets, large platelets contain more soluble factors which are responsible for stronger platelet aggregation and higher function of thrombosis formation. Indeed, studies have shown that reticulate platelets and platelets with larger volume have strong platelet activity and are predictors of cardiovascular risk^19–21^. Thoracotomy, acute hemorrhage, combining the loss of function of platelets in the circulation induced by clopidogrel might promote a consequent compensatory production of platelets that are larger and more reactive by the bone marrow in rats^22^. Grosdidier et al.^11^ demonstrated that the metabolism of both clopidogrel and prasugrel to their active form depended on CYP2C19; however, CYP2C19 loss-of-function alleles had no significant effect on the antiplatelet effects of prasugrel. There were few reports of prasugrel resistance and ticagrelor resistance, another antagonist of the platelet P2Y12 receptor. Thus, the pharmacodynamics of clopidogrel should be affected by some circulating factors in vivo.

TMAO is produced from TMA, which is a waste product of gut microbes that is oxidized by FMO3 in the liver. A recent study revealed that TMAO could enhance platelet responsiveness and activation by promoting Ca2+ release from intracellular stores in platelets resulting from the induction of ADP^13^. TMAO increased the potential for thrombosis in a carotid artery injury model in mice. Clinical studies further confirmed that TMAO could worsen arterial thrombosis risk (heart attack, stroke and mortality) in a dose-dependent manner^13,22–24^. Microbial transplantation of human gut commensals into gnotobiotic mice could also enhance platelet reactivity and thrombosis potential by increasing circulating TMAO levels^25^. Meanwhile, suppressing circulating TMAO levels by inhibiting FMO3 activity or adding analogs of choline (the main source of TMA), such as 3,3-dimethyl-1-butanol (DMB), iodomethylcholine (IMC) and fluoromethylcholine (FMC), significantly reduced platelet hyperresponsiveness and thrombosis risk^12,26,27^. Thus, TMAO plays an important role in inducing platelet hyperresponsiveness and increasing thrombosis risk. The present study revealed that TMAO could sufficiently suppress the inhibitory effects of clopidogrel on platelets (Fig. 3), induce CR (2/6), reduce the tail bleeding time (Fig. 2) and increase the myocardial thrombosis burden (Fig. 4). A previous study demonstrated that patients with coronary artery disease have higher circulating TMAO levels^14^. Every 10 µmol/L increase in TMAO predicted a 7.6% increase in all-cause mortality^17^. The failure of antiplatelet therapy with clopidogrel might be a major cause. In addition, several underlying causes of CR, such as obesity, long-term medications, stress, kidney disfunction trended to induce a higher TMAO level^28–30^. Based on the above findings, we speculate that the pharmacodynamics of clopidogrel are affected by circulating TMAO levels and that clopidogrel resistance is associated with elevated serum TMAO levels in patients with CAD.

Although the exact receptor and mechanisms by which TMAO activates platelets have not been clarified, Zhu et al.^13^ demonstrated that TMAO could induce calcium release from stores in platelets and activated platelets by altering the inositol 1,4,5-triphosphate (IP3) signaling pathway, which is a downstream signaling pathway of the G protein coupled receptor (GPCR)-phospholipase C (PLC) signaling pathway. ADP activated platelets via platelet P2Y1 and P2Y12 receptors, both of which are GPCRs. The P2Y1 receptor could alter the PLC-IP3 signaling pathway, while P2Y12 tended to alter cAMP expression when activated. The P2Y12 receptor plays a central role in sustaining platelet activation in response to ADP^31^. Zhu et al.^13^ suggested that TMAO could directly enhance platelet responsiveness to ADP. The present study revealed that TMAO could suppress the effects of the P2Y12 receptor inhibitor clopidogrel. Thus, TMAO and clopidogrel might act on the same receptor, or their effects may be mediated partially by the same mechanism. However, whether P2Y12 or G protein-coupled receptors are receptors for TMAO and the exact underlying mechanism warrant further investigation.

In conclusion, the present study demonstrated that circulating TMAO could suppress the inhibitory effects of clopidogrel on platelet aggregation. TMAO may be a potential mediator of clopidogrel resistance.

### Limitations

The findings of this study have to be seen in light of the following limitations. Firstly, we used the ischemia and reperfusion model in this study as a mimic of a most common type of coronary artery disease. However, the platelet reactivity of this model could not precisely represent that in coronary artery disease patients with anti-platelet therapy. Secondly, both prasugrel and ticagrelor are antagonist of the platelet P2Y12 receptor. Further studies are needed in order to compare the effects of TMAO between clopidogrel and prasugrel or ticagrelor. Thirdly, in the present study, CR was defined as a decrease in the platelet aggregation rate induced by clopidogrel under ADP stimulation of at least 30% compared with the mean value in the IR group. Common and proven CR definitions consider platelet aggregation in light transmission aggregometry or VASP-levels.

## Data Availability

The datasets generated during and/or analyzed during the current study are available from the corresponding author on reasonable request.
